# Docosahexaenoic Acid Modulates Autophagy and Confers Neuronal Resilience under Hypoxia–Reoxygenation Stress

**DOI:** 10.1007/s12031-026-02512-1

**Published:** 2026-03-30

**Authors:** Francis Zamora, Manuel L. Montero, Jo-Wen Liu, Viet Hoang Dinh, Johnny D. Figueroa, Marino A. De León

**Affiliations:** Center for Health Disparities and Molecular Medicine, Department of Basic Sciences, Physiology Division, Loma Linda University Health School of Medicine, 11085 Campus Street, Mortensen Hall, Loma Linda, CA USA

**Keywords:** Docosahexaenoic acid (DHA), Autophagy, Hypoxia–reoxygenation injury, Ooxidative stress, Neuroprotection

## Abstract

Hypoxia dramatically worsens neuronal death after injuries and strokes, as neurons rely heavily on oxidative phosphorylation for energy. This study investigated the effects of docosahexaenoic acid (DHA) in reducing cell death in nerve cells. Neuron-like cells, nerve growth factor-differentiated PC12 (NGFDPC12) cell cultures, with or without DHA pretreatment, were exposed to hypoxic conditions (0.5% O₂) for 12 to 48 h, followed by reoxygenation. Results showed that hypoxia caused a significant increase in the stress-response gene HIF-1α, with mRNA levels rising 5.47- and 6-fold at 24 and 48 h, respectively, compared to normoxic controls. Similarly, the pro-apoptotic gene BNIP3 was elevated 3.37- and 2.9-fold at the same time points. After 24 h of hypoxia, cells showed significant reactive oxygen species (ROS) buildup and displayed apoptotic features. The expression of fatty acid-binding protein 5 (FABP5), which is involved in responding to oxidative stress, also increased under hypoxic conditions. Treatment with DHA or rapamycin significantly improved cell viability after hypoxic exposure. To determine whether DHA protects by activating autophagy, we measured the expression of critical autophagy-related genes Atg5, Atg7, and Atg12. Hypoxia suppressed Atg5 and Atg7 expression; however, DHA restored and markedly increased the levels of Atg5, Atg7, and Atg12 beyond those in normoxic conditions. Immunoblot analysis supported these findings, showing higher levels of phosphorylated Beclin-1 and conjugated LC3, two key autophagy markers. These findings support our prior research, suggesting that DHA’s neuroprotective effects during hypoxia may involve the activation of autophagy pathways, highlighting its potential as a therapeutic strategy.

**Impact Statement**

This study reveals that docosahexaenoic acid (DHA), an omega-3 fatty acid abundant in the brain, enhances neuronal resilience to hypoxia–reoxygenation injury by restoring autophagy function and limiting oxidative stress. These findings uncover a previously underappreciated role for DHA as a modulator of redox-sensitive autophagy pathways and suggest potential therapeutic strategies for ischemic and metabolic neurodegenerative disorders.

**Supplementary Information:**

The online version contains supplementary material available at 10.1007/s12031-026-02512-1.

## Highlights

DHA pretreatment protects neuronal-like cells from hypoxia–reoxygenation injury by reducing oxidative stress and apoptosis.

Hypoxia suppresses autophagy-related genes, *Atg5* and *Atg7*, whereas DHA stimulates *Atg5*, *Atg7*, *Atg12* mRNA expression and enhances Beclin-1 phosphorylation and LC3 lipidation, indicating autophagy activation.

**Supplementary Information:**

The online version contains supplementary material available at 10.1007/s12031-026-02512-1.

## Introduction

The central nervous system (CNS) depends primarily on oxidative phosphorylation for energy production. During ischemia or hypoxia, this dependency renders neurons vulnerable to energy failure, leading to acidosis, glutamate excitotoxicity, and osmotic swelling (Dirnagl et al. 1999). Cells in the infarct core undergo necrosis, with the ischemic cascade extending to the penumbra (Radak et al. 2017). Subsequent processes involve oxidative stress, inflammation, and apoptosis (Kanekar et al. 2012). As post-mitotic cells, neurons cannot dilute toxins via division and rely heavily on autophagy for clearing damaged organelles and sustaining homeostasis (Maday 2016). Autophagy is crucial for neuronal survival and synaptic integrity, with neurons exhibiting greater dependence on basal autophagic flux than most cell types (Hara et al. 2006; Komatsu et al. 2006; Sidibe et al. 2022).

Docosahexaenoic acid (DHA; 22:6 n-3) is the predominant omega-3 polyunsaturated fatty acid in the CNS, comprising ~ 40% of polyunsaturated acyl chains in gray matter phospholipids (Lauritzen et al. 2000; Yehuda et al. 2002). Its multiple double bonds confer membrane fluidity, influencing biophysical properties and functional outcomes. DHA serves as precursor to specialized pro-resolving mediators (SPMs), including neuroprotectin D1, which mitigate inflammation and promote repair (Bazan 2005; Serhan and Petasis 2011). In hypoxic-ischemic models, DHA reduces infarct volume, neuronal loss, and oxidative stress while preserving mitochondrial function (Mayurasakorn et al. 2011). Notably, DHA promotes mitophagy—a selective autophagic process—to clear damaged mitochondria, alleviating ROS and supporting survival in PC12 cells under oxygen–glucose deprivation (Sun et al. 2022).

Our laboratory has adopted nerve growth factor differentiated rat pheochromocytoma 12 cells (NGFDPC12 cells) as an in vitro model to study neuronal injury. As a post-mitotic neuronal phenotype with neurites and hypoxia responsiveness, NGFDPC12 cells provide a validated model for studying ischemic injury and autophagic pathways (Greene and Tischler 1976; Medina-Pulido et al. 2013; Woronowicz et al. 2007). Our prior work showed DHA protects against lipotoxicity in NGFDPC12 cells and primary Schwann cells by maintaining lysosomal function, inhibiting mitochondrial permeabilization, and blocking apoptosis (Almaguel et al. 2010; Descorbeth et al. 2018; Montero et al. 2020). DHA also suppresses necroptosis and enhances autophagy, evidenced by LC3-II accumulation (Montero et al. 2020). Given hypoxia-reoxygenation induces ATP depletion, ROS, and calcium dysregulation, we posited DHA confers protection via autophagic activation. Here, we demonstrate DHA pretreatment boosts autophagic pathway in NGFDPC12 cells during 0.5% O₂ hypoxia followed by reoxygenation, with phosphorylated Beclin-1 and LC3-II as key markers. Elucidating this mechanism may inform omega-3-based therapies to attenuate ischemic neuronal damage.

## Materials and Methods

### Reagents and Materials

Ham’s F-12 medium with Kaighn’s modification (F-12 K; Mediatech, Inc., Manassas, VA) was used for all cell cultures. Horse serum and fetal bovine serum (Neuromics, Edina, MN) were supplemented as indicated. Human recombinant β-nerve growth factor (NGF; Thermo Fisher Scientific, Waltham, MA) was used for differentiation. Fatty acid-free bovine serum albumin (BSA; Cat# 126575-10GM, MilliporeSigma, Billerica, MA), docosahexaenoic acid (DHA; Cat# 90310, Cayman Chemical, Ann Arbor, MI), and rapamycin (Cat# 9904 S, Cell Signaling Technology, Danvers, MA) were used as described below. Halt protease & phosphatase inhibitor cocktail (Cat# 78441, Thermo Fisher Scientific) were included in all extraction buffers.

### Cell Culture and Differentiation

PC12 cells (rat pheochromocytoma; RRID: CVCL_0481, ATCC Cat# CRL-1721) were cultured on poly-L-lysine–coated six-well plates in F-12 K medium containing 15% horse serum, 2.5% fetal bovine serum (FBS), and 1% penicillin/streptomycin. Differentiation was initiated by switching to F-12 K medium supplemented with 50 ng/mL NGF, 1% FBS, and antibiotics (hereafter “1% FBS–NGF medium”). Medium was refreshed every 2–3 days, and cells were maintained for 7–10 days until extensive neurite outgrowth formed interconnected networks. Only fully differentiated cultures were used for experiments.

### Hypoxia–Reoxygenation Exposure

For hypoxia, NGFDPC12 cells in 1% FBS–NGF medium were placed in a Galaxy 48 R CO₂ incubator equipped with an O₂ controller (Eppendorf, Hauppauge, NY) maintained at 37 °C, 5% CO₂, and 0.5% O₂ (balanced with N₂). Normoxic controls were cultured at 37 °C, 5% CO₂, and ambient O₂ (~ 19.9%). Cells were subjected to hypoxia for 12–48 h followed by 12 h reoxygenation under normoxic conditions. Morphological changes were monitored by phase-contrast microscopy using an Olympus microscope with a SPOT-Insight CMOS camera.

### Fatty Acid Preparation and Treatment

DHA stocks (50 mM in ethanol) were freshly complexed with 150 µM fatty acid-free BSA in 1% FBS–NGF medium before use to make final fatty acid concentration of 50 µM (Clementi et al. 2019; Maralbashi et al. 2024; Montero et al. 2020; Zhang et al. 2018). BSA serves as fatty acid vehicle to ensure the concentration of unbound free fatty acids in the media is at constant level during incubation and the concentration of 150 µM, which is approximately 1% w/v, is within the range commonly used to prepare fatty acids solution for cell culture experiments (Ge et al. 2018; Ng and Say 2018; Ortiz-Rodriguez et al. 2019; Ricchi et al. 2009). For sets of experiments, NGF-differentiated PC12 cells were pretreated for 48 h with 50 µM DHA and then switched to 1% FBS–NGF medium before hypoxic exposure. During the fatty acid pretreatment, the control cells were incubated with 1% FBS–NGF medium containing 150 µM BSA and 0.1% ethanol.

### Quantitative Real-Time PCR (qRT-PCR)

Total RNA was extracted using TRI-Reagent (Molecular Research Center, Cincinnati, OH) and quantified spectrophotometrically (A260/A280 ≥ 1.9). cDNA was synthesized using the iScrip cDNA Synthesis kit (Bio-Rad Laboratories, Hercules, CA). qRT-PCR was performed using SYBR Green Master Mix (Bio-Rad) on a CFX96 Real-Time PCR Detection System (Bio-Rad). Primer specificity was verified by melt-curve analysis. Relative gene expression was calculated by the 2^-ΔΔCT method using β-actin as the reference gene and normoxic controls as calibrators. Primer sequences were as follows:

**Table:** 

Gene	Forward (5′→3′)	Reverse (5′→3′)
HIF-1α	TCCATGTGACCATGAGGAAA	CTTCCACGTTGCTGACTTGA
BNIP3	CCAGAAAATGTTCCCCCCAAG	TTGTCAGACGCCTTCCAATGTAG
FABP5	TTACCCTCGACGGCAACAA	CCATCAGCTGTGGTTTCATCA
β-actin	GGGAAATCGTGCGTGACATT	GCGGCAGTGGCCATCTC
ATG5	TGTCTCTGCTGTCCTGTTGG	GCAGCGAACTTCCCTTACTG
ATG7	CCCAAAGACATCAAGGGCTA	CCTGACTTTATGGCTTCCCA
ATG12	CGTCTTCGGTTGCAGTTTC	CCAGTTTACCATCACTGCCA

### Reactive Oxygen Species (ROS) Assay

Intracellular ROS were quantified by flow cytometry using 10 µM 2′,7′-dichlorodihydrofluorescein diacetate (H₂DCFDA; Invitrogen). After treatments, cells were incubated with dye for 25 min at 37 °C, detached with HyQtase (GE Healthcare), washed twice, and analyzed on a BD FACSCalibur flow cytometer (excitation = 488 nm; emission = 530 nm). Cells treated with 10 µM DL-buthionine-[S, R]-sulfoximine (BSO) for 24 h served as positive controls.

### Apoptosis Detection

Apoptosis was quantified using Annexin V–FITC staining. Following treatment, cells were detached with HyQtase, washed in binding buffer, and incubated with Annexin V–FITC for 20 min at room temperature in the dark. At least 10,000 events per sample were analyzed by flow cytometry, as previously described (Padilla et al. 2011).

### Cell Viability Assay

Cell viability was determined using the WST-1 assay (Roche Diagnostics). After treatments, medium was replaced with 2 mL of F-12 K containing 200 µL of WST-1 reagent per well. After 2 h incubation at 37 °C, absorbance was measured at 450 nm using a SpectraMax i3X spectrophotometer (Molecular Devices, Sunnyvale, CA). Blank readings (medium + reagent only) were subtracted, and data were expressed as a percentage of the control group.

### Immunoblotting

Total protein was extracted in Laemmli sample buffer (0.1 M Tris-HCl, pH 6.8, 4% SDS, 10% glycerol, protease & phosphatase inhibitors) and quantified by DC Protein Assay (Bio-Rad). Equal amounts of protein (15–40 µg) were separated on NuPAGE Bis-Tris gels (Invitrogen) and transferred to nitrocellulose membranes. Membranes were blocked with Intercept blocking buffer (Li-Cor Biotechnology, Lincoln, NE) and incubated overnight at 4 °C with primary antibodies against LC3A/B (Cat# 4108 S, CST) and phospho-Ser93-Beclin-1 (Cat# 14717 S, CST) and total Beclin-1 (Cat# 3495 S, CST). IRDye-labeled secondary antibodies were applied for detection on an Odyssey imaging system (Li-Cor Biotechnology). Band intensities were quantified using Image Studio 5.5 and normalized to β-actin.

### Statistical Analysis

All data are presented as mean ± SEM from at least three independent experiments and technical triplicate measurements were performed in WST-1 and real-time PCR assay. Statistical analyses were carried out in GraphPad Prism 9.4.1. Comparisons between two groups were performed using unpaired two-tailed Student’s t-tests. Multiple group comparisons employed one-way or two-way ANOVA followed by Tukey’s post-hoc test. Normality and variance homogeneity were verified prior to analysis. Statistical significance was accepted at *p* < 0.05.

## Results

### Hypoxia Induces Oxidative and Apoptotic Stress Responses in NGFDPC12 Cells

**Fig. 1:**
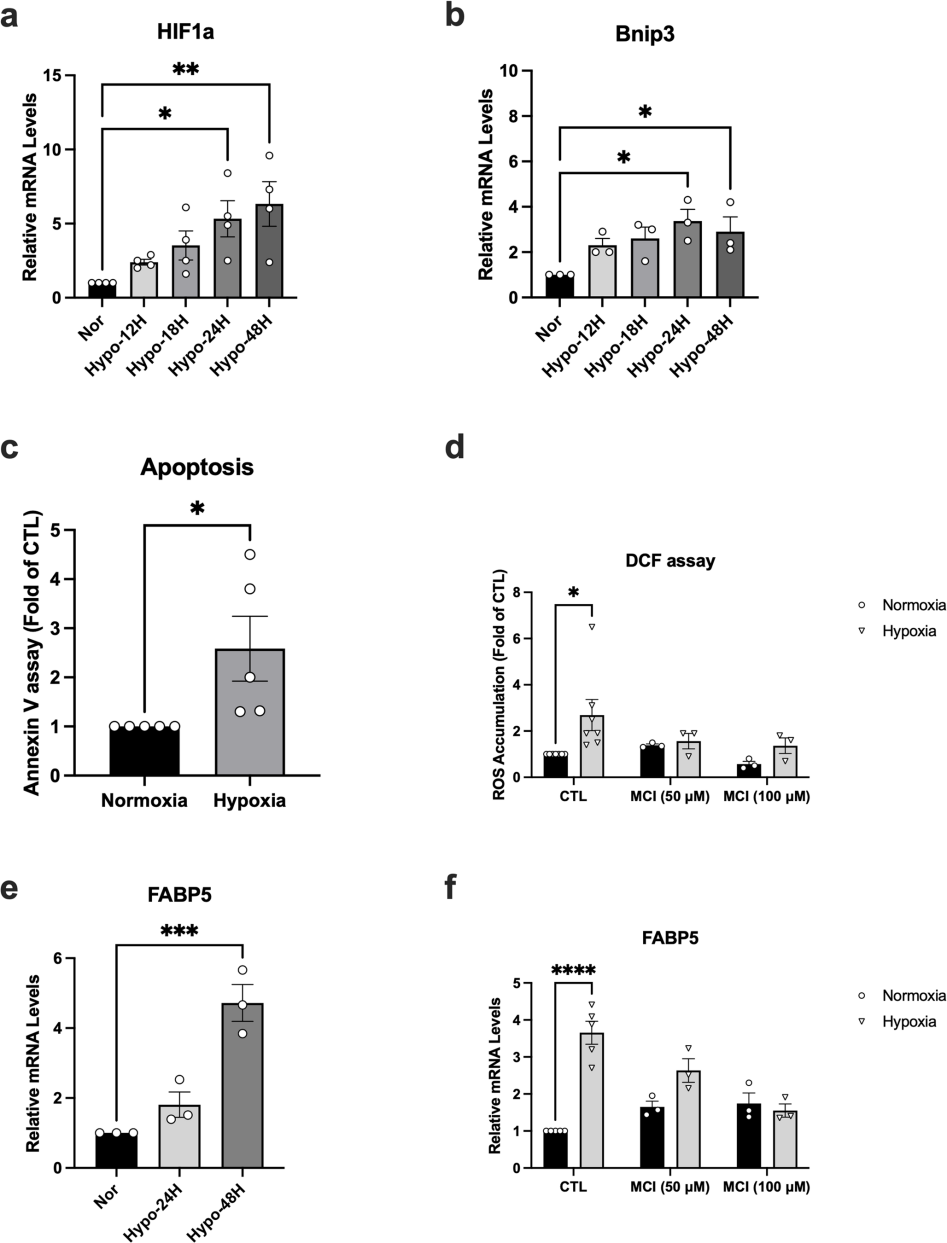
Hypoxia induces stress-related gene expression, ROS accumulation, and apoptosis in NGFDPC12 cells*.* Cells were exposed to 0.5% O₂ for 12–48 h. (**a**) *HIF-1α* and (**b**) *BNIP3* mRNA levels were quantified by qRT-PCR. (**c**) Annexin V–positive cells were measured by flow cytometry after 24 h of hypoxia. (**d**) Intracellular ROS after 24 h of hypoxia were analyzed using the DCF assay with or without antioxidant MCI-186 (50–100 µM). (**e**) *FABP5* expression after 24–48 h of hypoxia was measured by qRT-PCR. (**f**) *FABP5* expression after 48 h of hypoxia was measured by qRT-PCR in the presence or absence of antioxidant MCI-186. Data represent mean ± SEM (*n* ≥ 3). For panels A and B, each data point represents the mean of three technical replicate measurements obtained in a single independent experiment. *p < 0.05; **p < 0.01; ****p < 0.0001

Exposure of NGFDPC12 cells to 0.5% O₂ for up to 48 h significantly increased *HIF-1α* mRNA in a time-dependent manner (2.5 ± 0.3-fold at 12 h; 6.0 ± 0.7-fold at 48 h; *p* < 0.01) (Fig. 1a). Expression of the downstream target *BNIP3* similarly rose 2.3–3.4-fold (Fig. 1b). Hypoxia also elevated apoptosis and oxidative stress: Annexin V–positive cells increased 2.6-fold (Fig. 1c), and intracellular ROS rose 2.7-fold relative to normoxia (Fig. 1d). The antioxidant MCI-186 (50–100 µM) attenuated ROS levels to ~ 1.5-fold, confirming oxidative involvement. *FABP5*, a stress-response gene, was up-regulated during hypoxia and suppressed by MCI-186 (Fig. 1e and f). Together, these results establish that exposure 0.5% O₂ for more than 24 h serves as a reliable in vitro model of neuronal hypoxic stress.

### DHA and Rapamycin Protect NGFDPC12 Cells from Hypoxic Injury

**Fig. 2:**
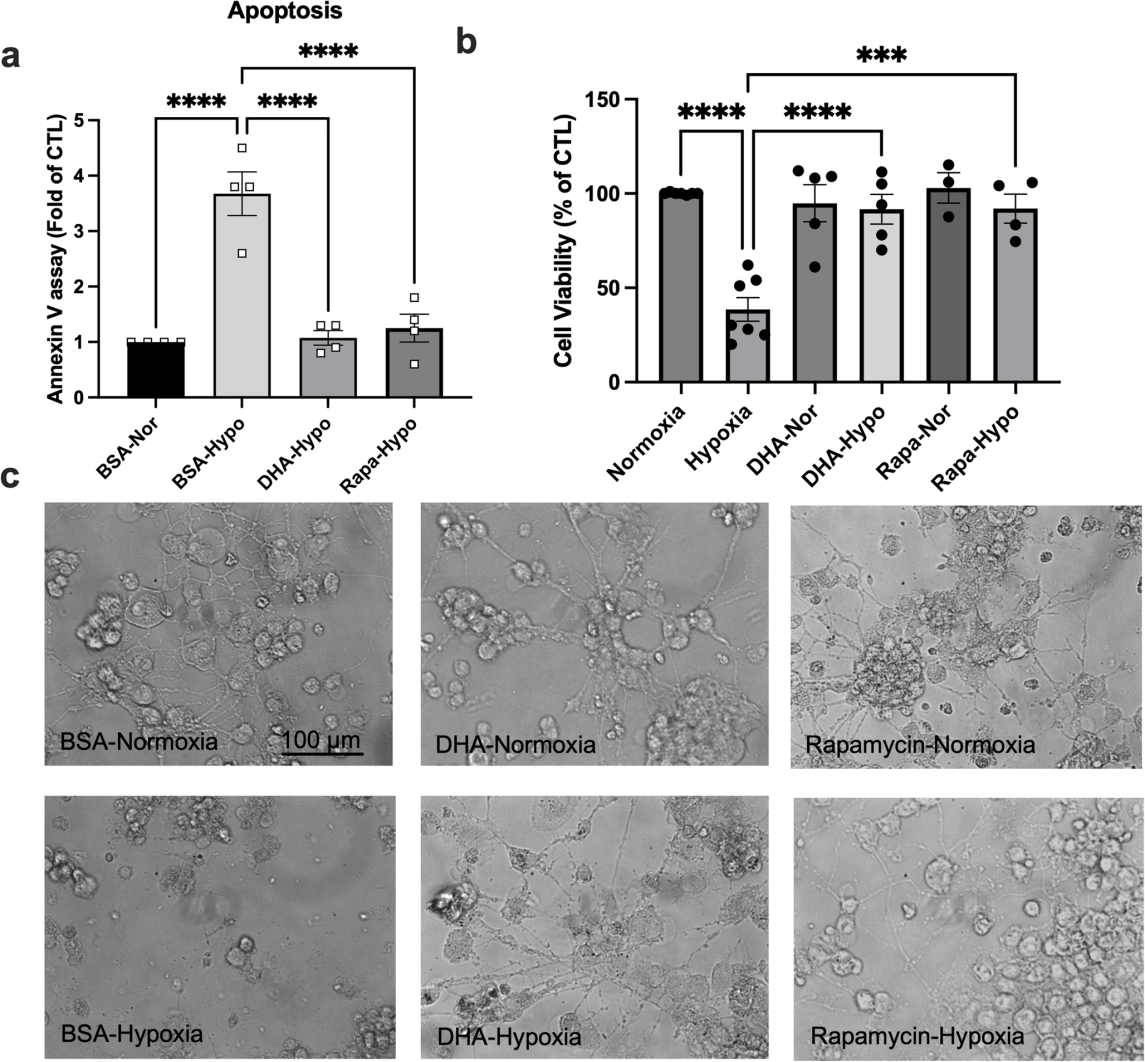
DHA and rapamycin protect NGFDPC12 cells from hypoxic injury. Cells were pretreated with DHA (50 µM) for 48 h or co-treated with rapamycin (100 nM) during hypoxia. (**a**) Apoptosis was assessed using the Annexin V assay after 24 h of hypoxia. (**b**) Cell viability was determined by the WST-1 assay after 48 h of hypoxia. (**c**) Representative phase-contrast images show cell morphology at 200x total magnification. Data = mean ± SEM (n ≥ 3); *p < 0.05; **p < 0.01; ***p < 0.001; ****p < 0.0001.

Pretreatment with 50 µM DHA or co-treatment with rapamycin significantly reduced hypoxia-induced apoptosis. Annexin V-positive cells decreased from 3.2 ± 0.3-fold (hypoxia) to 1.2 ± 0.2-fold with either treatment (*p* < 0.001) (Fig. 2a). WST-1 assays confirmed improved viability after 48 h hypoxia: 38.6 ± 4.2% (hypoxia) vs. 79.5 ± 5.0% (DHA) and 92 ± 4% (rapamycin) (*p* < 0.001) (Fig. 2b). Morphologically, DHA- and rapamycin-treated cells retained neurite structure and confluency compared with hypoxic controls (Fig. 2c), indicating preserved cellular integrity.

### DHA Restores Expression of Autophagy-Related Genes Suppressed by Hypoxia

**Fig. 3:**
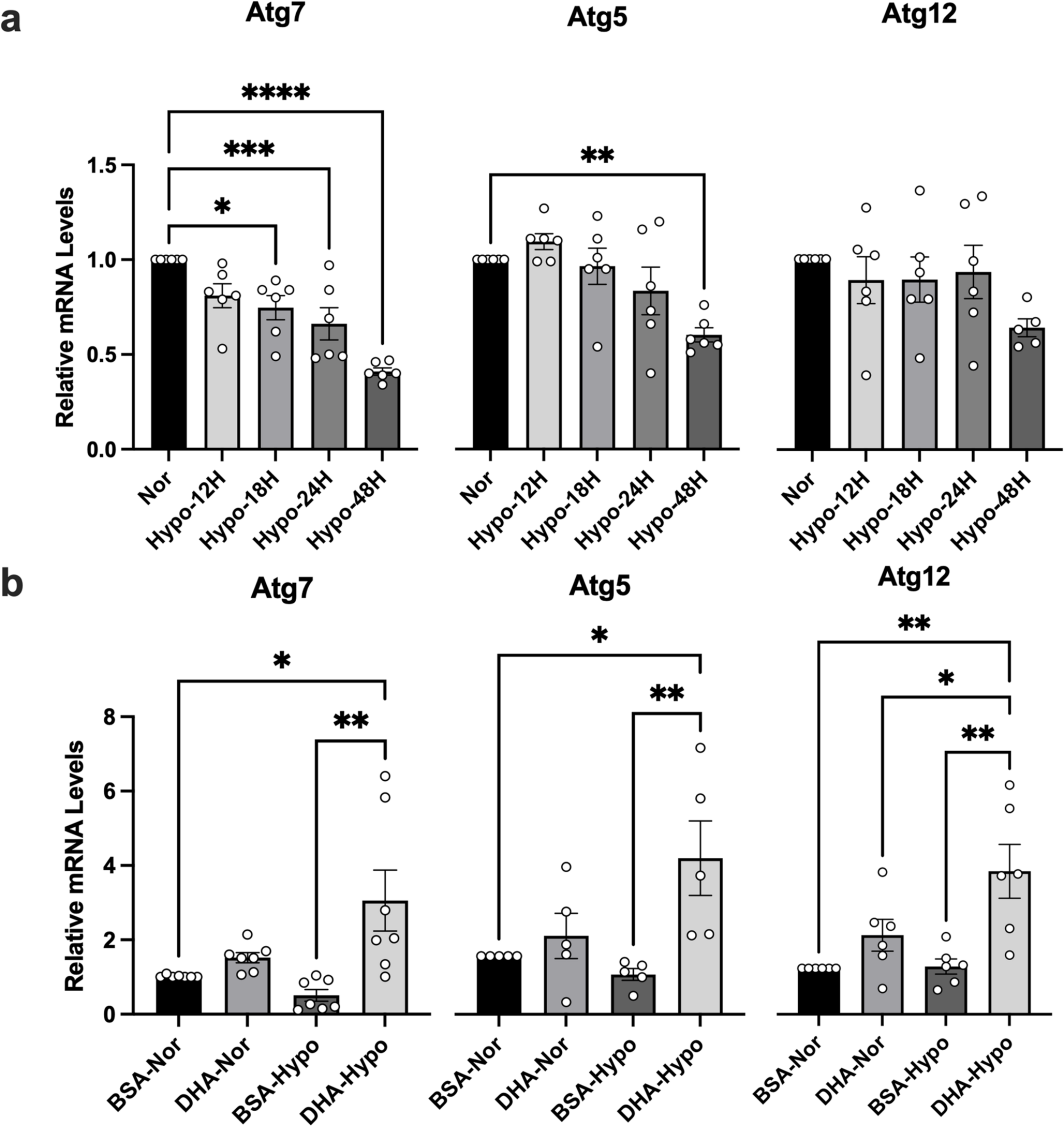
DHA restores autophagy-related gene expression suppressed by hypoxia. (**a**) Cells were exposed to 0.5% O₂ for 12–48 h. (**b**) Cells were pretreated with DHA (50 µM) or vehicle (BSA) for 48 h and then subjected to 48 h hypoxia. *Atg7*, *Atg5*, and *Atg12* mRNA levels were measured by qRT-PCR. Data = fold-change relative to normoxia (mean ± SEM, n ≥ 3). *p < 0.05; **p < 0.01; ***p < 0.001; ****p < 0.0001.

The *Atg7*,* Atg5* and *Atg12* mRNA levels exhibit a downward trend after hypoxia treatment. (Fig. 3a). A*tg7* transcripts were significantly reduced after 18 h and *Atg5* transcripts were significantly reduced at 48 h. DHA pretreatment counteracted this suppression, increasing expression of *Atg7* (3.0 ± 0.3-fold), *Atg5* (2.7 ± 0.4-fold), and *Atg12* (3.0 ± 0.5-fold) relative to normoxia (*p* < 0.001) (Fig. 3b). Thus, DHA enhances the autophagy machinery impaired by hypoxia.

### DHA Enhances Beclin-1 Phosphorylation and LC3 Lipidation Under Hypoxia

**Fig. 4:**
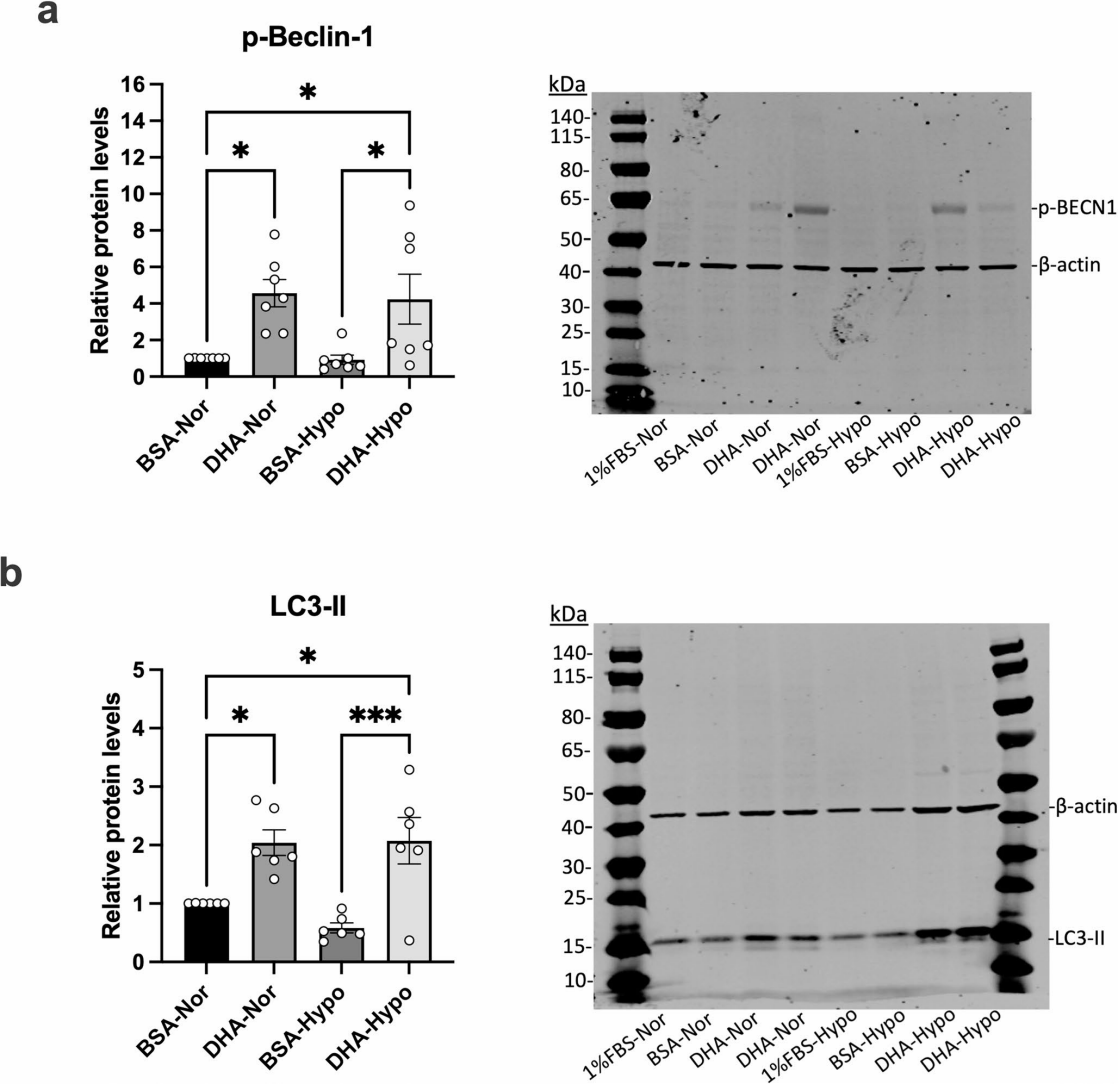
DHA enhances Beclin-1 phosphorylation and LC3 lipidation under hypoxia. Cells were pretreated with DHA (50 µM) or vehicle (BSA) and exposed to 0.5% O₂ for 24 h. (**a**) Beclin-1 (Ser93) phosphorylation and (**b**) LC3-II levels were analyzed by Western blot. Representative blots and densitometric quantifications are shown. Data = fold-change relative to normoxia (mean ± SEM, n ≥ 3). *p < 0.05; ***p < 0.001.

Western blot analyses revealed that hypoxia reduced phospho-Beclin-1 (Ser93) to 0.5 ± 0.1-fold of control (*p* < 0.05), while DHA increased it 4.6 ± 0.4-fold in normoxia and 4.2 ± 0.5-fold in hypoxia (*p* < 0.01) (Fig. 4a). Total Beclin-1 levels remained unchanged across experimental conditions (Supplemental Fig. 1), reinforcing the proposed mechanistic framework. LC3-II levels also fell under hypoxia (0.58 ± 0.08-fold) but rose to ~ 2.0 ± 0.3-fold with DHA (Fig. 4b). These results demonstrate that DHA activates Beclin-1–dependent autophagy signaling and autophagosome formation even under hypoxic conditions.

### Summary

**Fig. 5:**
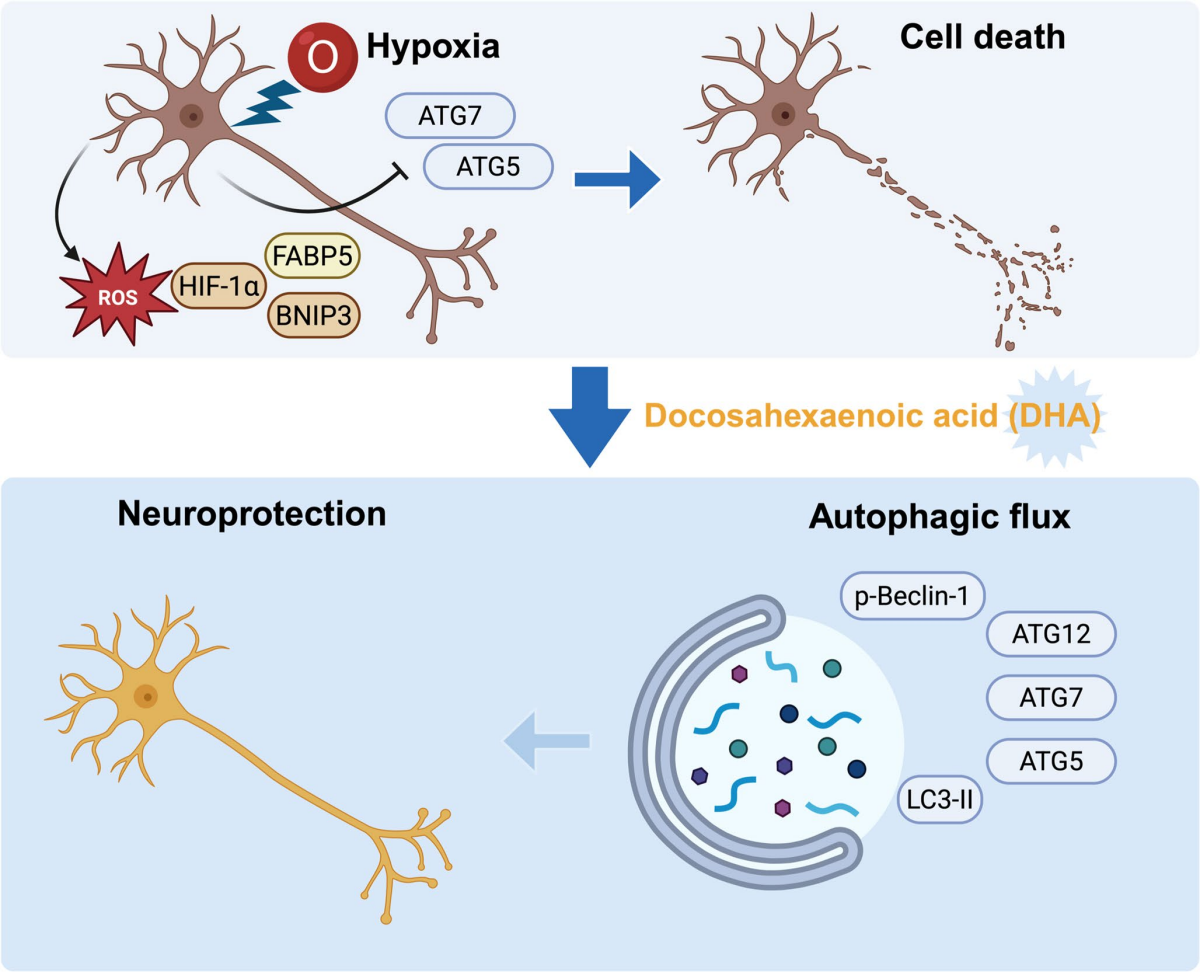
Proposed mechanism of DHA-induced neuroprotection under hypoxia–reoxygenation stress. Hypoxia exacerbates neuronal injury by impairing oxidative phosphorylation and triggering oxidative stress, apoptosis, and suppression of autophagy. In this study, nerve growth factor-differentiated PC12 (NGFDPC12) cells exposed to 0.5% O₂ showed increased expression of the stress-response genes *HIF-1α* and *BNIP3*, accumulation of reactive oxygen species (ROS), and upregulation of the oxidative stress marker *FABP5*. Pretreatment with docosahexaenoic acid (DHA) or the autophagy activator rapamycin markedly improved cell viability and reduced apoptosis. DHA restored the expression of autophagy-related genes (*Atg5*, *Atg7*, *Atg12*) suppressed by hypoxia and increased Beclin-1 phosphorylation and LC3 lipidation, consistent with enhanced autophagic activity. Together, these findings support a model in which DHA pretreatment induces mild ROS signaling that activates the AMPK–Beclin-1–LC3 pathway, promoting autophagy and reducing oxidative stress and apoptosis under hypoxia–reoxygenation stress. The illustration was generated with BioRender.com

DHA mitigates hypoxia-induced apoptosis and oxidative stress by restoring autophagy gene expression and stimulating Beclin-1/LC3 signaling. Parallel protection by rapamycin supports autophagy induction as a key mechanism of DHA-mediated neuroprotection (Fig. 5).

## Discussion

This study identifies DHA as a powerful enhancer of autophagy that protects neuronal-like cells from hypoxic injury. Hypoxia (0.5% O₂) activates the canonical HIF-1α/BNIP3 pathway, increases ROS production, and triggers apoptosis in NGFDPC12 cells. DHA pretreatment restores autophagic gene expression, enhances Beclin-1 phosphorylation, and increases LC3 lipidation, thereby improving cell survival. Under normal oxygen levels, mitochondrial ROS are neutralized by antioxidant enzymes (Droge 2002). During hypoxia, ATP depletion weakens these defenses, and upon reoxygenation, NADPH oxidase activation further boosts ROS production (He et al. 2023; Trebak et al. 2010). Consistent with this mechanism, MCI-186 effectively reduces hypoxia-induced ROS in our model. The simultaneous upregulation of FABP5—a ROS-responsive lipid chaperone (Liu et al. 2015)—further confirms the oxidative stress in hypoxic NGFDPC12 cells.

Autophagy and apoptosis share molecular components and often act antagonistically to determine cell fate (Bellot et al. 2009; Gu et al. 2015). BNIP3, induced by HIF-1α, controls this cross-talk by releasing Beclin-1 from Bcl-2/Bcl-xL inhibition, thus activating autophagy (Li et al. 2021; Zhang and Ney 2009). In our experiments, hypoxia suppressed Beclin-1 phosphorylation and LC3-II formation, indicating impaired autophagy. DHA restored both, suggesting a shift toward autophagic survival signaling. The upregulation of Atg5, Atg7, and Atg12 further supports DHA’s role in maintaining the autophagic machinery during metabolic stress. Interestingly, DHA increased Beclin-1 phosphorylation and LC3-II even under normoxia, implying that it primes autophagy. DHA has been shown to activate AMPK in neural tissues (Datilo et al. 2023), which phosphorylates Beclin-1 at Ser93/96 to stimulate autophagy initiation. We propose that DHA generates a mild redox signal that engages AMPK–Beclin-1 pathways, establishing a preconditioned state that enhances resilience to subsequent hypoxic stress. Low-level ROS production can serve as a beneficial signal for homeostatic adaptation (Miller et al. 2019; Redza-Dutordoir et al. 2016), supporting this hypothesis.

Although the experiments reported in this study were conducted in differentiated PC12 cells, which do not form glutamatergic synapses that are important mediators of neuronal injury under hypoxia/reperfusion conditions, the cellular mechanisms identified may still have clinical relevance. In diabetic neuropathy, chronic hyperglycemia and microvascular dysfunction lead to reduced endoneurial oxygenation and axonal degeneration (McMillan 1984; Nukada 2014). Structural changes in the microvasculature—such as basement-membrane thickening, pericyte loss, and endothelial proliferation—hinder oxygen delivery (Cho et al. 2008; Taylor 2001), making diabetic nerves more susceptible to ischemic injury (Nukada et al. 2011; Wang et al. 2004). Our prior clinical studies showed that DHA supplementation alleviates neuropathic pain and decreases circulating markers of oxidative and necroptotic stress (Duran et al. 2019, 2022). The current findings support these observations mechanistically by showing that DHA enhances autophagic flux and reduces hypoxia-induced cell death. In conclusion, DHA restores autophagic capacity, boosts Beclin-1 activation, and decreases oxidative and apoptotic stress in neuronal-like cells. These results highlight autophagy induction as a key mechanism of DHA-supported neuroprotection, endorsing the therapeutic potential of ω-3 fatty acids in ischemic and metabolic neuropathies. Future research involving autophagic flux reporters, AMPK inhibitors, and in vivo models of cerebral or peripheral ischemia is essential to further elucidate this pathway. Accordingly, future studies should confirm these findings in primary neuronal cultures and, ideally, in vivo models to enhance their physiological relevance.

## Supplementary Information

Below is the link to the electronic supplementary material.

Supplementary Material 1 (DOCX 315 KB)

## Data Availability

The datasets generated and/or analyzed during the current study are available from the corresponding author upon reasonable request.
