# Supplementary material for: Docosahexaenoic Acid Modulates Autophagy and Confers Neuronal Resilience under Hypoxia–Reoxygenation Stress
Source: J Mol Neurosci. 2026 Mar 30;76(2):56. doi: 10.1007/s12031-026-02512-1 (PMC13035634; doi:10.1007/s12031-026-02512-1)
Supplement: Supplementary file 1 — Supplementary Material 1 (DOCX 315 KB) [file 12031_2026_2512_MOESM1_ESM.docx]

**Supplemental Figure 1. *Total Beclin-1 levels are not affected by DHA in NGFDPC12 cells*. Cells were pretreated with DHA (50 μM) or vehicle (BSA) and then exposed to 0.5% O₂ for 24 hours. Total Beclin-1 levels were measured using Western blot. Densitometric quantification (a) and representative immunoblots for anti-BCN1 (b) and anti-β-actin (c) show that total Beclin-1 remains unchanged across conditions. Data are expressed as fold-change relative to normoxia (mean ± SEM, n=3). *p* > 0.05.**
